# Emergence, spread, and impact of high‐pathogenicity avian influenza H5 in wild birds and mammals of South America and Antarctica

**DOI:** 10.1111/cobi.70052

**Published:** 2025-05-31

**Authors:** Thijs Kuiken, Ralph Eric Thijl Vanstreels, Ashley Banyard, Lineke Begeman, Andrew C. Breed, Meagan Dewar, Ruben Fijn, Patricia Pereira Serafini, Marcela Uhart, Michelle Wille

**Affiliations:** ^1^ Department of Viroscience Erasmus University Medical Centre Rotterdam The Netherlands; ^2^ Karen C. Drayer Wildlife Health Center, School of Veterinary Medicine University of California, Davis Davis California USA; ^3^ Influenza and Avian Virology Workgroup, Department of Virology Animal and Plant Health Agency Weybridge UK; ^4^ WOAH/FAO International Reference Laboratory for Avian Influenza Animal and Plant Health Agency Weybridge UK; ^5^ Epidemiology, Surveillance and Laboratory Section, Department of Agriculture, Fisheries and Forestry Australian Government Canberra Australian Capital Territory Australia; ^6^ Future Regions Research Centre Federation University Australia Berwick Victoria Australia; ^7^ Bird Ecology Department Waardenburg Ecology Culemborg The Netherlands; ^8^ Laboratório de Biomarcadores de Contaminação Aquática e Imunoquímica Universidade Federal de Santa Catarina Florianópolis Brazil; ^9^ Centre for Pathogen Genomics, Department of Microbiology and Immunology at the Peter Doherty Institute for Infection and Immunology The University of Melbourne Melbourne Victoria Australia

**Keywords:** Antarctica, avian influenza, emerging infectious diseases, marine mammals, one health, seabirds, South America, virology, Antártida, aves marinas, enfermedades infecciosas emergentes, gripe aviar, mamíferos marinos, Sudamérica, una sola salud, virología

## Abstract

The currently circulating high‐pathogenicity avian influenza (HPAI) virus of the subtype H5 causes variable illness and death in wild and domestic birds and mammals, as well as in humans. This virus evolved from the Goose/Guangdong lineage of the HPAI H5 virus, which emerged in commercial poultry in China in 1996, spilled over into wild birds, and spread through Asia, Europe, Africa, and North America by 2021. Our objective was to summarize the spread and impact of the HPAI H5 virus in wild birds and mammals in South America, evaluate the risk of its spread and potential impact on Antarctic wildlife, and consider actions to manage the current and future HPAI outbreaks in wildlife. We obtained data on HPAI H5 virus detection and reported wildlife deaths from websites, newspaper articles, and scientific publications. The virus arrived in South America in October 2022. Thereafter, it spread widely and rapidly throughout the continent, where it infected at least 83 wild bird species and 11 wild mammal species and is estimated to have killed at least 667,000 wild birds and 52,000 wild mammals. The HPAI H5 virus spread to the Antarctic region by October 2023 and to mainland Antarctica by December 2023. This spread was associated with multiple mortality events in seabirds and marine mammals. The high spatial density of colonies of various Antarctic species of birds and mammals provides conditions for potentially devastating outbreaks with severe conservation implications. Ecosystem‐level impacts may follow, and affected populations may take decades to recover. Although little can be done to stop the virus spread in wildlife, it is important to continue targeted surveillance of wildlife populations for HPAI H5 virus incursion and assessment of the spread and impact of disease to inform adaptation of conservation plans and to help policy makers mitigate and prevent future HPAI outbreaks.

## Abstract (es)

Surgimiento, propagación e impacto de la gripe aviar H5 de alta patogenicidad en aves y mamíferos silvestre de Sudamérica y la Antártida

**Resumen:**

El virus de la gripe aviar de alta patogenicidad (HPAI) del subtipo H5 que circula actualmente causa enfermedades variables y la muerte de aves y mamíferos silvestres y domésticos, y también de humanos. Este virus evolucionó a partir del linaje Goose/Guangdong del virus HPAI H5, que apareció en aves de corral comerciales en China en 1996, se extendió a las aves silvestres y se propagó por Asia, Europa, África y Norteamérica en 2021. Nuestros objetivos son resumir la propagación y el impacto del virus HPAI H5 en aves silvestres y mamíferos en Sudamérica, evaluar el riesgo de su propagación y el impacto potencial en la fauna silvestre antártica y considerar acciones para gestionar los brotes actuales y futuros de HPAI en la fauna silvestre. Obtuvimos datos sobre la detección del virus HPAI H5 y sobre las muertes de animales silvestres reportadas en sitios web, artículos de prensa y publicaciones científicas. El virus llegó a Sudamérica en octubre de 2022. A partir de entonces se propagó amplia y rápidamente por todo el continente, donde infectó al menos a 83 especies de aves silvestres y 11 especies de mamíferos silvestres y se estima que mató al menos a 667,000 aves silvestres y 52,000 mamíferos silvestres. El virus HPAI H5 se propagó a la región antártica en octubre de 2023 y a la Antártida continental dos meses después. Esta propagación se asoció a múltiples eventos de mortalidad en aves y mamíferos marinos. La alta densidad espacial de las colonias de varias especies antárticas de aves y mamíferos proporciona las condiciones para brotes potencialmente devastadores con graves implicaciones para la conservación. Los impactos pueden producirse a nivel de ecosistema y las poblaciones afectadas pueden tardar décadas en recuperarse. Aunque se puede hacer poco para detener la propagación del virus en la fauna salvaje, es importante continuar la vigilancia específica de las poblaciones de fauna salvaje para detectar la incursión del virus HPAI H5 y la evaluación de la propagación y el impacto de la enfermedad para fundamentar la adaptación de los planes de conservación y ayudar a los políticos responsables a mitigar y prevenir futuros brotes de gripe aviar de alta patogenicidad.

## INTRODUCTION

The continued emergence of the high‐pathogenicity avian influenza (HPAI) virus of the H5 subtype is a major hazard for wildlife health and conservation. In contrast to most infectious disease agents, the HPAI H5 virus has a broad host range among wild birds and mammals, is highly infectious, and can cause high mortality. The HPAI H5 virus therefore represents a novel but poorly understood threat to a broad range of wild animal populations and their ecosystems. This has been exemplified in South America, where the HPAI H5 virus had not been reported until 2022 and thus entered into wild bird and mammal populations that presumably had never been exposed to that virus. It spread rapidly across the continent in the following months, causing mass mortality events of unprecedented magnitude in many wildlife species, in particular seabirds and marine mammals (Ariyama et al., 2023; Campagna et al., 2023; Carrazco‐Montalvo et al., 2023; Cruz et al., 2023; Leguia et al., 2023; Marandino et al., 2023).

The current epidemic HPAI H5 virus is a descendant of the A/Goose/Guangdong/1/96 (Gs/GD) lineage. The term *high pathogenicity* refers to the high morbidity and mortality rates in infected chickens, not necessarily in other infected species. The term *H5* refers to the number of the hemagglutinin subtype to which this virus belongs; avian influenza viruses are categorized into 16 subtypes based on the antigenic properties of their hemagglutinin (Neumann et al., 2021). Although mortality from other HPAI viruses is mainly restricted to poultry, the Gs/GD lineage is unusual in also causing mortality in wild birds and mammals. Since its emergence, HPAI H5 has caused mortality in at least 356 species of wild birds (Klaassen & Wille, 2023a), 49 species of wild mammals (European Food Safety Authority et al., 2023), and hundreds of millions of poultry (Shi et al., 2023). It also has caused mortality in hundreds of people (Lai et al., 2016), with case fatality rates varying among clades, countries, periods, and types of exposure. Transmission among a wide variety of species demonstrates the interconnectedness of domestic animals, wildlife, humans, and their shared environment and highlights the need for a one‐health approach to HPAI H5. The consensus definition of the *one health approach* was formulated in 2021 by the interdisciplinary One Health High‐Level Expert Panel and supported by the United Nations Food and Agriculture Organization, the World Organization for Animal Health, the United Nations Environment Programme, and the World Health Organization: “an integrated, unifying approach that aims to sustainably balance and optimize the health of people, animals, and ecosystems. It recognizes that the health of humans, domestic and wild animals, plants, and the wider environment (including ecosystems) are closely linked and interdependent” (One Health High‐Level Expert Panel et al., 2022; World Health Organization, 2021).

The Gs/GD lineage of the HPAI H5 virus was first detected in commercially farmed geese in China in 1996 and has circulated and evolved in poultry since then (Xie et al., 2023). Multiple virus variants in the Gs/GD lineage spread among the rapidly growing poultry populations in Asia. In 2005, there was a substantial spillover into migratory wild birds and subsequent spread to Europe and Africa. The virus caused numerous outbreaks in wild birds in Asia, Europe, and Africa in the following years, typically during autumn and winter, with spread primarily linked to migratory movements of wild birds. Additional resurgent events occurred in 2014, 2016, and 2020 that were associated with the emergence of the 2.3.4.4 clade (Xie et al., 2023). Since 2021, however, one clade of the HPAI H5 virus (2.3.4.4b) has persisted year‐round in wild birds in Europe (Pohlmann et al., 2022). This clade of the HPAI H5 virus (hereafter HPAI H5 virus for the pathogen and HPAI H5 for the associated disease) spread across the Atlantic (in 2021) and Pacific Oceans in 2022 to North America (Alkie et al., 2022; Caliendo et al., 2022), where it spread rapidly across the continent during 2022 and spread southward, reaching Central and South America by October 2022 (European Food Safety Authority et al., 2022), South Georgia (*Islas Georgia del Sur*) by October 2023 (Banyard et al., 2024), and the Antarctic Peninsula by February 2024 (Scientific Committee of Antarctic Research, 2024).

Although national and international surveillance for HPAI H5 provides a relatively accurate overview of the geographic spread of HPAI H5 and its impact on poultry populations, as well as the occurrence of human infections, surveillance and mortality estimates are limited in wild birds and mammals (Klaassen & Wille, 2023b). Hence, the picture of the spread and impact of HPAI H5 in wildlife is fragmented across numerous reports and notifications (e.g., Caliendo et al., 2024; Camphuysen & Gear, 2022). We here define *impact* as a major effect on affected wildlife species, including loss of individual lives; disruption of social structures, such as those between parents and offspring; and reduction of population numbers. Also, there is little evidence by which routes HPAI H5 can spread through the Antarctic region because many wildlife species in the region are unique to that continent and their movements through the region are poorly understood (Bestley et al., 2020; Shirihai et al., 2008). Furthermore, the potential impact on Antarctic wildlife populations is unclear because exposure risks and species susceptibility are poorly known. Therefore, we sought to synthesize data on the spread of the HPAI H5 virus and, as a measure of its impact, of associated mortality in wildlife in South America and the Antarctic region; to evaluate potential pathways for further introduction and virus spread through the Antarctic region; and to review potential concerns for wildlife conservation of HPAI H5 emergence in South America and Antarctica. By focusing on the impact of HPAI H5 on wildlife and ecosystems, which have been relatively neglected compared with the impacts on poultry and people (Klaassen & Wille, 2023b; Kuiken et al., 2005), we sought to support greater equity among the health of ecosystems, wild and domestic animals, and humans and so emphasize the one health approach regarding HPAI H5 (One Health High‐Level Expert Panel et al., 2022).

## DATA

We obtained data on HPAI H5 virus detection and reported wildlife deaths in Central America and South America from reports to the World Organization for Animal Health (WOAH), which are centrally archived on the website of the World Animal Health Information System (WAHIS). These data were supplemented by data from the national government websites of Argentina (Argentina Ministerio de Economía Secretaría de Agricultura Ganadería y Pesca, 2023a, 2023b; Argentina Servicio Nacional de Sanidad y Calidad Agroalimentaria, 2023), Brazil (Brazil Ministério da Agricultura e Pecuária, 2023a, 2023b), and Chile (Chile Servicio Agrícola y Ganadero, 2023; Chile Servicio Nacional de Pesca y Acuicultura, 2023); newspaper articles (Brazil, Uruguay); and scientific publications (Argentina, Brazil). Data for the Falkland Islands (*Islas Malvinas*) were obtained from a national government website (Falkland Islands Department of Agriculture, 2024) and a newspaper article (Mercopress, 2024). Data for South Georgia were obtained from a scientific publication and the website of the Agreement on Conservation of Albatrosses and Petrels. Data for the Antarctic Peninsula were obtained from the website of the Scientific Committee for Antarctic Research (Scientific Committee on Antarctic Research, 2024). Data on the numbers of individuals found dead per species and country are in Appendix S1. The HPAI H5 virus infection was confirmed in all reported species–country associations, with a few exceptions, as specified below.

## SPREAD OF HPAI H5 THROUGH SOUTH AMERICA FROM OCTOBER 2022 TO DECEMBER 2023

**FIGURE 1:**
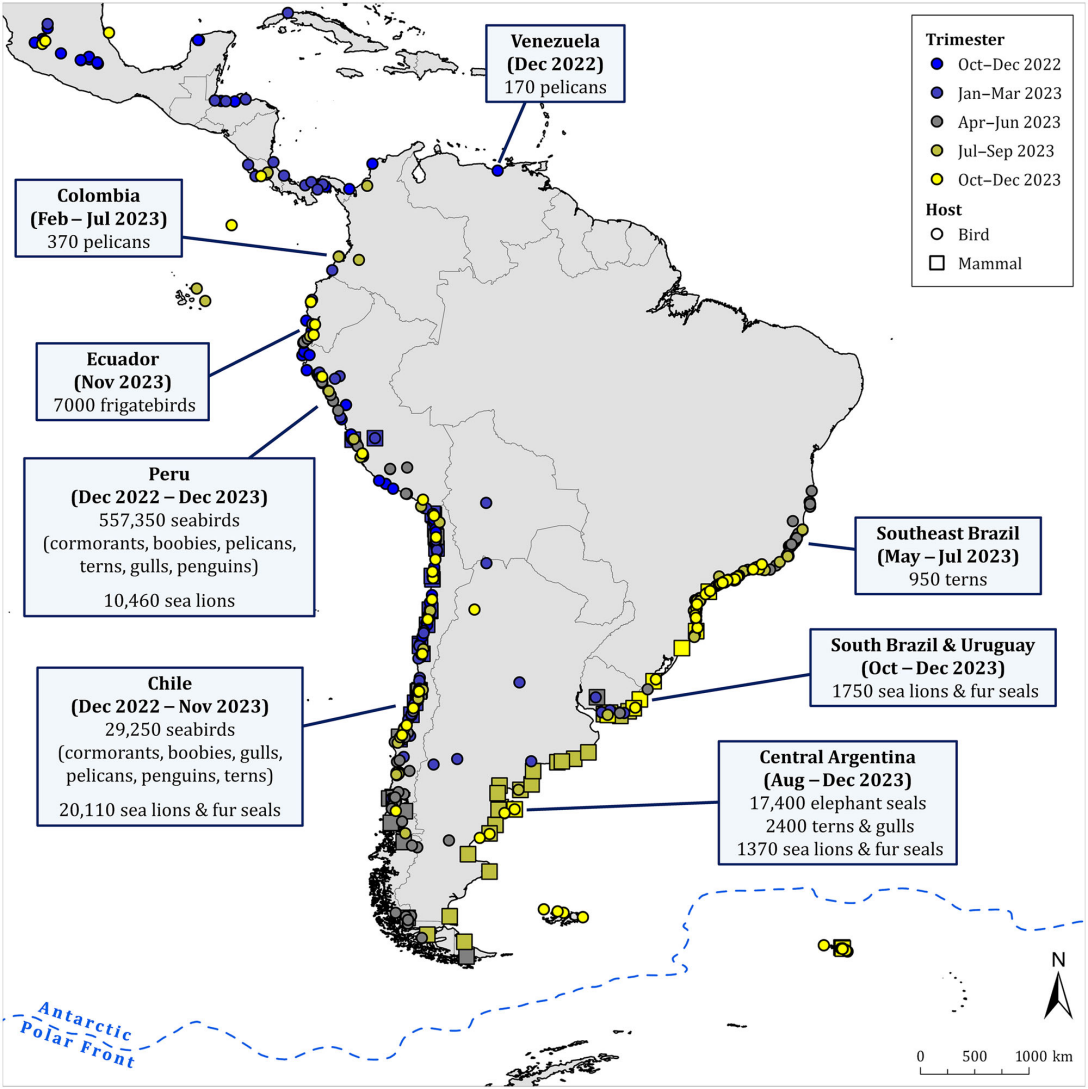
Locations of reported mortalities associated with high‐pathogenicity avian influenza virus of the subtype H5 in wild birds and wild mammals in Central America, South America, and neighboring island groups (Galápagos, Falkland [Malvinas], South Georgia [Georgia del Sur]) from October 2022 to December 2023 (text boxes, selected major mortality events). Detailed of mortality reports are in Appendix S1.

In the year following its introduction to South America, the HPAI H5 virus infected at least 83 wild bird species and 11 wild mammal species and was the probable cause of death of at least 667,000 wild birds and 52,000 wild mammals (Figure 1; Appendix S1). For ease of reading, mortality counts >10 are rounded off to the nearest tenth, hundredth, or thousandth, depending on the scale of mortality. Precise counts of dead animals are in Appendix S1. These data were derived from reports of HPAI H5 virus detections in wild animals found ill or dead and mortality counts of wild animals during HPAI H5 outbreaks. Numerical comparisons among countries may be unreliable because countries differ considerably in their approach to surveillance, diagnostic methods, and reporting of suspected or confirmed HPAI cases.

The correlation between the distribution of wildlife cases reported and human population density strongly suggests the potential for vast underreporting in sparsely populated areas (Klaassen & Wille, 2023a). Therefore, these data need to be interpreted with caution. For most species affected (e.g., pelicans, boobies, cormorants, sea lions), there was robust evidence that the unusual mortality recorded in South America during 2022–2023 was largely attributable to HPAI H5, with evidence including the high frequency of HPAI H5 detection in carcasses, spatiotemporal patterns of morbidity and mortality consistent with the epidemiology of an acute highly transmissible disease, and the absence of other factors that could explain the mortality events. However, there were a few species (e.g., shearwaters, ibises, parakeets) for which the evidence was less robust, and it is plausible that other causes of mortality may have coincided with HPAI H5 outbreaks. Regarding the scale of mortality events, it is extremely unlikely that all sick or dead animals were found and reported, especially in remote areas where observations and surveillance efforts were scarce. For example, collection rates of waterbird carcasses during typical avian botulism outbreaks in the North American prairie are 10–25% of total mortality (Bollinger et al., 2011). Moreover, because the outbreaks extended over months and then seasons, surveillance, detection, and testing efforts markedly decreased in the region, with some countries in South America failing to report new wildlife cases since late 2023. Therefore, the actual levels of wildlife mortality from HPAI H5 were undoubtedly much higher than the reported counts.

**FIGURE 2:**
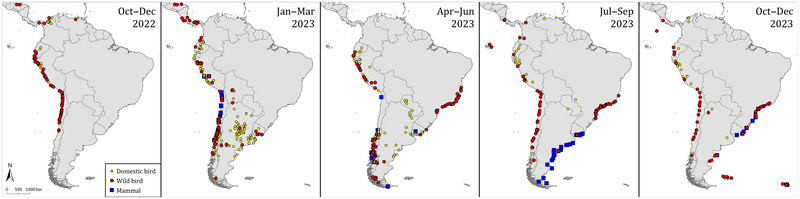
Progression per trimester of reported mortalities associated with high‐pathogenicity avian influenza virus of the subtype H5 in wild birds, wild mammals, and domestic birds in Central America, South America, and neighboring island groups (Galápagos, Falkland [Malvinas], South Georgia [Georgia del Sur]) from October 2022 to December 2023.

The chronology of HPAI H5 virus detections and associated wildlife mortalities, combined with genetic analyses (Banyard et al., 2024; Jimenez‐Bluhm et al., 2023; Leguia et al., 2023; Marandino et al., 2023; Pardo‐Roa et al., 2025; Reischak et al., 2023; Rimondi et al., 2024), suggests that the HPAI H5 virus entered South America in October 2022 and that multiple independent viral introduction events occurred (Cruz et al., 2023; Leguia et al., 2023). Not all introduced viruses spread further through the continent. Following the virus introduction, HPAI H5 spread southward along the west coast of South America (Peru and Chile) from November 2022 to January 2023. It subsequently spread via 2 separate pathways. First, it spread eastward across the Andes to infect poultry and waterbirds on the La Plata Basin (Bolivia, northern Argentina, Uruguay, and Paraguay), inland across central and southern Argentina from February to April 2023, and to eastern Brazil's seabirds on the Atlantic coast from June to October 2023. The second pathway was southward along the southern Pacific coast (Chile), where it infected seabirds and marine mammals (Castro‐Sanguinetti et al., 2024), to the southern tip of the continent, and subsequently northward along the Atlantic coast (Argentina, Uruguay, and southern Brazil) from August to December 2023 (Figure 2).

### Venezuela November 2022

One of the first outbreaks of HPAI H5 in wildlife occurred in November 2022 in Venezuela (WAHIS, 2022c) (Figure 1), where it affected 200 brown pelicans (*Pelecanus occidentalis*) (WAHIS, 2022d). This die‐off in Venezuela is consistent with the relatively low mortality of wild birds reported in Central America in December 2022 and January 2023, specifically in Panama (Promed mail, 2022), Honduras (WAHIS, 2022b), Costa Rica (WAHIS, 2023f), and Guatemala (WAHIS, 2023h). Overall, numbers of wild birds reported dead in these countries were relatively low (hundreds) and mostly restricted to brown pelicans.

### Peru December 2022 to December 2023

The largest HPAI‐H5‐associated mass mortality event occurred along the coast of Peru. Large numbers of seabirds and marine mammals that feed on abundant fish populations inhabiting the nutrient‐rich Peruvian current upwelling ecosystem were affected (Figure 1) (Peru Ministerio de Salud, 2023). About 558,000 seabirds of at least 14 species were found dead, mainly from December 2022 to June 2023 (Appendix S1; Figure 2). This number represents 84% of the total number of wild birds found dead in South America. The most frequently recorded species were cormorants (Phalacrocoracidae spp.) (*n* = 255,000), Peruvian boobies (*Sula variegate*) (*n* = 236,000), Peruvian pelicans (*Pelecanus thagus*) (*n* = 58,000), Inca terns (*Larosterna inca*) (*n* = 8000), and gulls (Larinae spp.) (*n* = 1000). In addition, 11,000 South American sea lions (*Otaria byronia*) were found dead, mainly from January to March 2023. A short‐beaked common dolphin (*Delphinus delphis*) also was found dead during that period.

### Chile December 2022 to November 2023

There was HPAI‐H5‐associated mass mortality of aquatic birds—both sea and freshwater—and marine mammals along the coast of Chile, part of the same Peruvian current upwelling ecosystem as in Peru (Figure 1) (Chile Servicio Agrícola y Ganadero, 2023; Chile Servicio Nacional de Pesca y Acuicultura, 2023). About 97,000 aquatic birds of at least 46 species were found dead, mainly from December 2022 to June 2023 (Appendix S1; Figure 2). The main species found dead were guanay cormorants (*Leucocarbo bougainvilliorum*) (*n* = 30,000), sooty shearwaters (*Ardenna grisea*) (*n* = 24,000), Peruvian boobies (*n* = 13,000), kelp gulls (*Larus dominicanus*) (*n* = 6000), Peruvian pelicans (*n* = 6000), Humboldt penguins (*Spheniscus humboldti*) (*n* = 5000), gray gulls (*Larus modestus*) (*n* = 4000), Neotropical cormorants (*Nannopterum brasilianum*) (*n* = 1000), black‐necked swans (*Cygnus melancoryphus*) (*n* = 1000), and elegant terns (*Thalasseus elegans*) (*n* = 800). In addition to aquatic birds, HPAI H5 was also detected in 9 species of terrestrial birds, with 1000 individuals found dead, mainly turkey vultures (*Cathartes aura*) (*n* = 600).

There also were 22,000 marine mammals of 9 species found dead, mainly in the period from January to June 2023, starting a month later than reported seabird mortality (Chile Servicio Agrícola y Ganadero, 2023; Chile Servicio Nacional de Pesca y Acuicultura, 2023). This number represents 41% of the total number of wild mammals found dead in South America in association with this HPAI H5 outbreak. The main species affected was the South American sea lion (*n* = 21,000); their mortality was more than double that recorded in Peru. Other marine mammal species affected included short‐beaked common dolphins (*n* = 60), marine otters (*Lontra felina*) (*n* = 50), Burmeister's porpoises (*Phocoena spinipinnis*) (*n* = 40), South American fur seals (*Arctocephalus australis*) (*n* = 40), and Chilean dolphins (*Cephalorhynchus eutropia*) (*n* = 20).

### Bolivia, Argentina, Paraguay, Uruguay, and Brazil January to June 2023

Starting in January 2023, a number of HPAI H5 outbreaks were recorded in poultry and wild waterbirds along the Rio de la Plata basin across Bolivia (WAHIS, 2023b), Argentina (initially at the north, then spreading southward to the Patagonian steppe) (Argentina Servicio Nacional de Sanidad y Calidad Agroalimentaria, 2023; WAHIS, 2023a), Paraguay (WAHIS, 2023i), Uruguay (WAHIS, 2023j), and southern Brazil (Brazil Ministério da Agricultura e Pecuária, 2023a, 2023b; Reischak et al., 2023; WAHIS, 2023c). Most detections in wild waterbirds were not accompanied by large‐scale mortality, with the exception of isolated clusters of mortality of black‐necked swans (*Cygnus melancoryphus*) (*n* = 300) in southern Brazil, Uruguay, and Argentina. By early May 2023, most of these outbreaks had resolved and HPAI H5 detections dwindled. In mid‐May 2023, a new wave of HPAI H5 detections emerged in seabirds in Espirito Santo, eastern Brazil. Cayenne terns (*Thalasseus acuflavidus eurygnathus*), royal terns (*Thalasseus maximus*), and South American terns (*Sterna hirundinacea*) were most heavily affected, with over 1000 birds found dead among all 3 species. Although most of the seabird mortality occurred in May and June 2023 and was concentrated in Espirito Santo, HPAI H5 was detected sporadically in various species of seabirds found ashore (ill or dead) throughout the southeastern and southern coast of Brazil over the following months, suggesting continued HPAI H5 circulation in seabirds in the region.

### Colombia February 2023

In February 2023, a brown pelican die‐off in Colombia (Figure 1) on Gorgona Island near the Pacific coast occurred; 400 individuals died (WAHIS, 2022a). This is the only mass mortality event reported in Colombia thus far.

### Coastal Argentina August to December 2023

There was HPAI‐H5‐associated mass mortality of marine mammals and later of seabirds along the coast of Argentina (Figure 1) at the edge of the Patagonian Shelf, which extends from 35° S latitude south to the tip of Tierra del Fuego and from the coast to approximately the 1000 m isobath (Falabella et al., 2009; Raya Rey & Huettmann, 2020). From August to December, there were 19,000 marine mammals of 3 species found dead along the Atlantic coast of Argentina (Appendix S1; Figure 2). Although small numbers of South American sea lions were found dead in the south of Argentina in August 2023 (Argentina Ministerio de Economía Secretaría de Agricultura Ganadería y Pesca, 2023a, 2023b), mortality soon spread north along the country's entire coast in August to December 2023. More than 1000 South American sea lions and South American fur seals were found dead at a few monitored sites (Argentina Ministerio de Economía Secretaría de Agricultura Ganadería y Pesca, 2023a, 2023b). In October 2023, there was a mass mortality of southern elephant seal pups (*Mirounga leonina*) attributed to HPAI H5 in Peninsula Valdés, Chubut province. It was estimated that 97% of young died (over 17,000 deaths), the largest mortality event recorded for this species (Campagna et al., 2023; Uhart et al., 2024).

From October to November 2023, 3000 seabirds of at least 10 species were found dead along the shoreline. Terns were the most heavily affected; 2000 individuals in total of South American, Cayenne, and royal terns died at a few monitored sites along the coast of Patagonia (Argentina Servicio Nacional de Sanidad y Calidad Agroalimentaria, 2023). Because most tern breeding sites in this region were not monitored during this period, the overall mortality was likely higher.

### Uruguay and southern Brazil October to December 2023

Shortly after the mortality of pinnipeds in Argentina, an HPAI‐H5‐associated mass mortality of more than 1000 South American sea lions and South American fur seals (available reports fail to distinguish between the 2 species) occurred along the coasts of Uruguay (El Observador, 2023a) and southern Brazil (Figures 1 & 2; Appendix S1) (Vara & Mano, 2023), simultaneous with the mortality of pinnipeds recorded along the coast of Argentina.

### Ecuador November 2023

Although HPAI H5 outbreaks were reported in Ecuadorian poultry since December 2022, detections in wildlife were relatively scarce and not associated with large mortalities. In November 2023, however, magnificent frigatebirds (*Fregata magnificens*) (*n* = 6000) and great frigatebirds (*Fregata minor*) (*n* = 1000) were found dead in association with HPAI H5 virus infection in areas of mangrove on the Ecuadorian coast (WAHIS, 2023g). These deaths were preceded in September 2023 by the detection of HPAI H5 in a small number of great frigatebirds and red‐footed boobies (*Sula sula*) on the Galápagos Islands (1000 km west of mainland Ecuador) (WAHIS, 2023g). Fortunately, these earlier detections at the Galápagos were not associated with large wildlife mortality events.

## RISK OF FURTHER HPAI H5 SPREAD IN THE ANTARCTIC REGION

**FIGURE 3:**
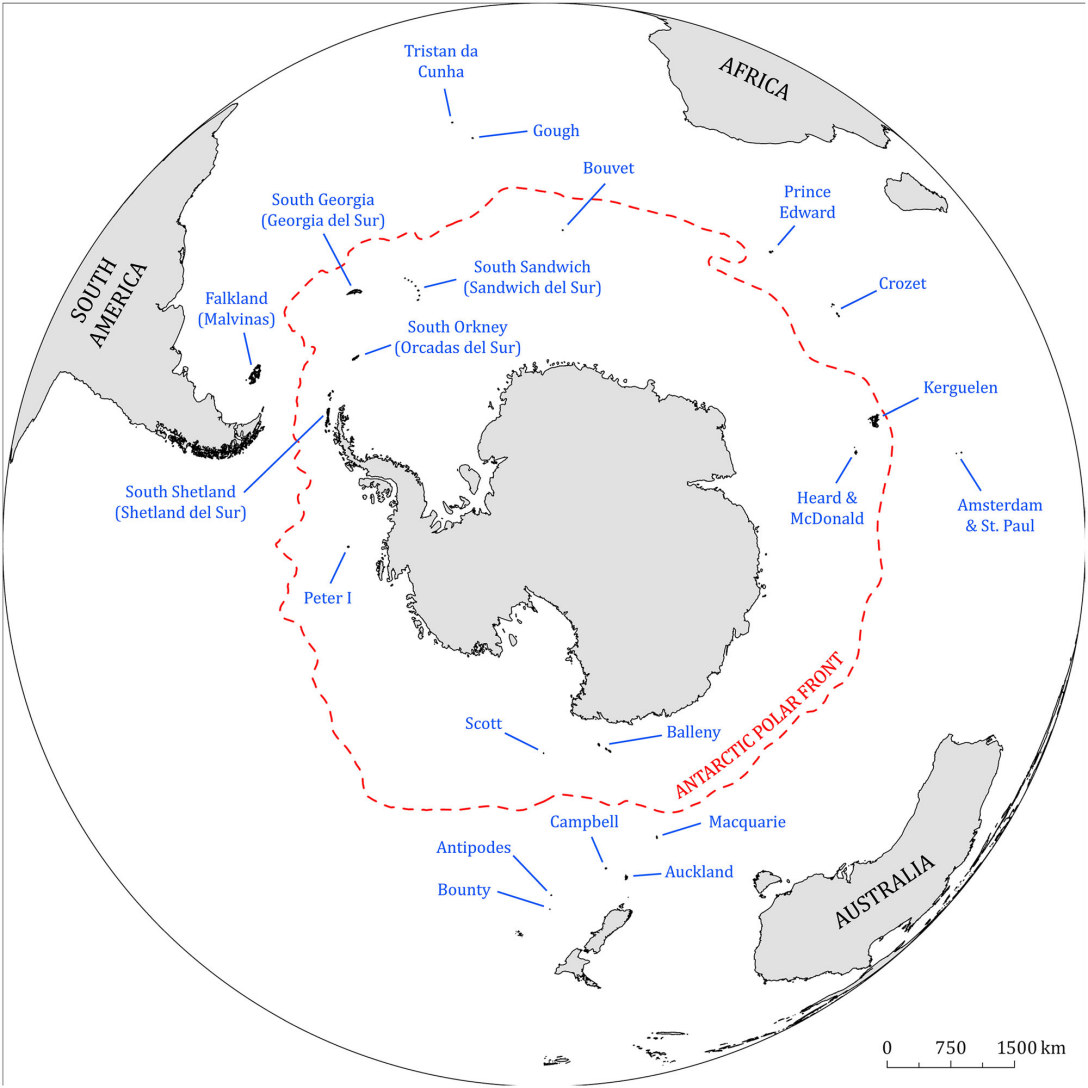
Approximate position of the Antarctic Polar Front (dashed red line) redrawn from Moore et al. (1999) and sub‐Antarctic island groups.

Following the rapid southbound movement of HPAI H5 through South America, its spread through Antarctica is highly plausible. Geographically, the area of Antarctica is not uniformly defined. Provisions of the Antarctic Treaty apply to the area south of latitude 60° S (Secretariat of the Antarctic Treaty, 2023). However, biogeographically, the Antarctic region extends north of this line and includes the waters and islands up to the Antarctic polar front, where colder and nutrient‐rich southern waters meet warmer northern waters (Figure 3). The Antarctic polar front is also the boundary of the area of the Convention for the Conservation of Antarctic Marine Living Resources (CCAMLR, 2023). We use the Antarctic polar front as the boundary of the Antarctic region. This definition excludes the Falkland, Gough, Prince Edward, Crozet, Amsterdam–St Paul, Macquarie, Auckland, Campbell, and Bounty and Antipodes islands, which we consider here as part of the sub‐Antarctic region when discussing the spread and impacts of HPAI H5.

### Spread of HPAI H5 to the Falkland Islands and South Georgia, October to December 2023

The Falkland Islands are 1000 km east of the mainland coast of Argentina and are part of the Patagonian Shelf. In October and November 2023, around the same time as seabird and marine mammal die‐offs were reported along the coasts of Argentina and Uruguay (see above), HPAI H5 was detected in 2 southern fulmars (*Fulmarus glacialoides*), and one black‐browed albatross (*Thalassarche melanophris*) found dead on the Falkland Islands (Falkland Islands Department of Agriculture, 2024). In December 2023, there was a mortality of approximately 30 black‐browed albatrosses attributed to HPAI H5 (Falkland Islands Department of Agriculture, 2024).

South Georgia is an archipelago in the Antarctic Polar Front and lies 1000 km southeast of the Falkland Islands. South Georgia is part of the Scotia Arc, a chain of islands between the southern tip of South America and the Antarctic Peninsula, which also includes the South Sandwich Islands (*Islas Sándwich del Sur*), the South Orkney Islands (*Islas Orcadas del Sur*), and the South Shetland Islands (*Islas Shetland del Sur*). In October and November 2023, about 60 brown skuas (*Stercorarius antarcticus*), 20 kelp gulls, 1 Antarctic tern (*Sterna vittata*), and unspecified numbers of southern elephant seals and Antarctic fur seals (*Arctocephalus gazella*) were found dead in association with HPAI H5 at multiple locations on South Georgia (Scientific Committee on Antarctic Research, 2023). Based on a limited number of publicly available virus sequences, genetic analysis showed the HPAI H5 virus from Bird Island to be most closely related to those from Uruguay, Peru, and Chile collected from December 2022 to April 2023 (Banyard et al., 2024; Government of South Georgia and the South Sandwich Islands, 2023; Scientific Committee on Antarctic Research, 2023).

### Significant recent events in the Antarctic region, January to March 2024

The HPAI‐H5‐associated deaths of more than 200 gentoo penguin (*Pygoscelis papua*) chicks and thousands of black‐browed albatross chicks on the Falkland Islands were recorded in January 2024 (Mercopress, 2024). Brown skuas and a variable hawk (*Geranoaetus polyosoma*) were found dead in January and February 2024 (Scientific Committee of Antarctic Research, 2024). In February 2024, the mortality of more than 50 adult wandering albatrosses (*Diomedea exulans*) in South Georgia was attributed to HPAI H5 (ACAP, 2024; Falkland Islands Department of Agriculture, 2024).

Furthermore, the HPAI H5 virus was detected in 2 skuas found dead in February 2024 on Primavera Cape, on the west coast of the Antarctic Peninsula, and in 5 skuas found dead on James Ross Island, off the northeast coast of the Antarctic Peninsula (Falkland Islands Department of Agriculture, 2024). It is unclear whether these were brown skuas or south polar skuas (*Stercorarius maccormicki*). These were the first detections of HPAI H5 on the continent of Antarctica (Scientific Committee of Antarctic Research, 2024).

### Potential pathways for further introduction and spread of HPAI H5 in the Antarctic region

There are 3 main potential pathways through which additional incursion events into the Antarctic region could occur: introduction to the Antarctic Peninsula by migratory species foraging on the Patagonian Shelf; introduction to the Antarctic region by native Antarctic species overwintering throughout the austral temperate zone; and introduction to the Antarctic region by transequatorial migratory species. The Patagonian Shelf may be the most probable and frequent source for the spread of the HPAI H5 virus to the Antarctic region, with the Falkland Islands and South Georgia likely playing a role as stepping stones. Given that more than 60 species of resident and visiting seabirds forage on the Patagonian Shelf (Croxall & Wood, 2002; Falabella et al., 2009; Quintana, 2002; Raya Rey & Huettmann, 2020; Salyuk et al., 2022; Zamora et al., 2023), there are myriad scenarios by which seabirds may spread the virus further in the Antarctic region. Preliminary evidence from virus genome sequence analysis suggests that seabirds, such as southern fulmars and brown skuas, in which HPAI H5 was detected on the Falkland Islands and South Georgia, may have been infected on the Patagonian Shelf (Banyard et al., 2024). This is also supported by the numerous detections of the HPAI H5 virus in seabirds in Argentina and Uruguay in preceding months (Argentina Servicio Nacional de Sanidad y Calidad Agroalimentaria, 2023; Brazil Ministério da Agricultura e Pecuária, 2023b; WAHIS, 2023c). Seabirds could transport the HPAI H5 virus when migrating southward to their breeding areas in the Scotia Arc, the Antarctic Peninsula, and Antarctic and sub‐Antarctic islands. In addition to seabirds, marine mammals also should be considered as possible vectors of the HPAI H5 virus, assuming that some individuals may be infected without showing clinical signs. Marine mammals may swim long distances during the period of infection, which in seals is about 1 week (Webster et al., 1981). For example, southern elephant seals leave their breeding sites after breeding or molting and migrate south to Antarctica to feed on squid and fish at the edge of the sea ice (Lewis et al., 2006; McGovern et al., 2022; Rodriguez et al., 2017). This implies that, from the Peninsula Valdés or South Georgia, infected southern elephant seals could conceivably transport the virus to the remainder of the Scotia Arc and to the Antarctic Peninsula. Further, marine mammals that have succumbed to HPAI H5 may represent a source of infection for avian species that feed on their carcasses.

Species that are native to the Antarctic region could be exposed to HPAI H5 while foraging in other areas in the austral temperate zone. For instance, Antarctic prions (*Pachyptila desolata*), the most southerly breeding of all prion species, could play a role in virus spread and amplification. In an individual found dead on the southeast coast of Brazil in September 2023, HPAI H5 was detected. This species breeds in large numbers on islands of the Scotia Arc, sub‐Antarctic islands in the Indian Ocean near New Zealand, and probably on Scott Island near the Antarctic continent. They congregate in large rafts at sea just before dusk and attend the colonies in huge flocks just after dark, which would provide opportunities for HPAI H5 transmission. After the breeding season, Antarctic prions disperse in a wide geographical range between the Antarctic pack‐ice and about 35° S. They are commonly found both on the Patagonian Shelf as well as in the Humboldt Current off South America during the austral winter (Navarro et al., 2015; Quillfeldt et al., 2013). Similarly, during the austral winter, other seabirds such as large numbers of various petrel species may also forage diffusely on open waters and continental shelves of the austral temperate zone and could be exposed to HPAI H5 through at‐sea interactions with other subtropical and temperate species. The susceptibility of petrel species is known from sporadic HPAI H5 detections in southern giant petrels (*Macronectes giganteus*) found ashore in Chile, northern giant petrels (*Macronectes halli*), and great‐winged petrels (*Pterodroma macroptera*) in Chile and South Africa and white‐chinned petrels (*Procellaria aequinoctialis*) in Brazil (Appendix S1).

The HPAI H5 virus could be introduced to the Antarctic region by transequatorial migratory species such as Arctic terns (*Sterna paradisaea*), long‐tailed jaegers (*Stercorarius longicaudus*), or south polar skuas. For example, the south polar skua spends the austral winter (boreal summer) in the North Pacific and North Atlantic and breeds in relatively snow‐free areas of Antarctica in the austral summer. Although this species is usually reliant on fish, in some areas it can rely solely on predation or scavenging of penguins (Kopp et al., 2011), which could provide opportunities for transmission of HPAI H5 viruses acquired in the Northern Hemisphere.

Following introduction to Antarctica, there will likely be numerous opportunities for HPAI H5 virus spread in this region. The Scotia Arc and the Antarctic Peninsula are home to large colonies of seabirds (especially penguins), Antarctic fur seals, and southern elephant seals, which are known or likely to be susceptible to HPAI H5 (Appendix S2). Low‐pathogenicity avian influenza strains (subtypes H4N7, H5N5, H6N8, and H11N2) have been detected in Adélie (*Pygoscelis adeliae*), gentoo, and chinstrap penguins (*Pygoscelis antarcticus*); southern giant petrels; snowy sheathbills (*Chionis albus*); and brown skuas, and phylogenetic analyses indicate virus circulation in the Antarctic over several years (Barriga et al., 2016; de Seixas et al., 2022; de Souza Petersen et al., 2017; Hurt et al., 2014, 2016; Ogrzewalska et al., 2022). This indicates that these species are suitable hosts for the transmission and persistence of influenza viruses and suggests that, if introduced, HPAI H5 strains potentially could spread and cause significant impacts on these populations.

**FIGURE 4:**
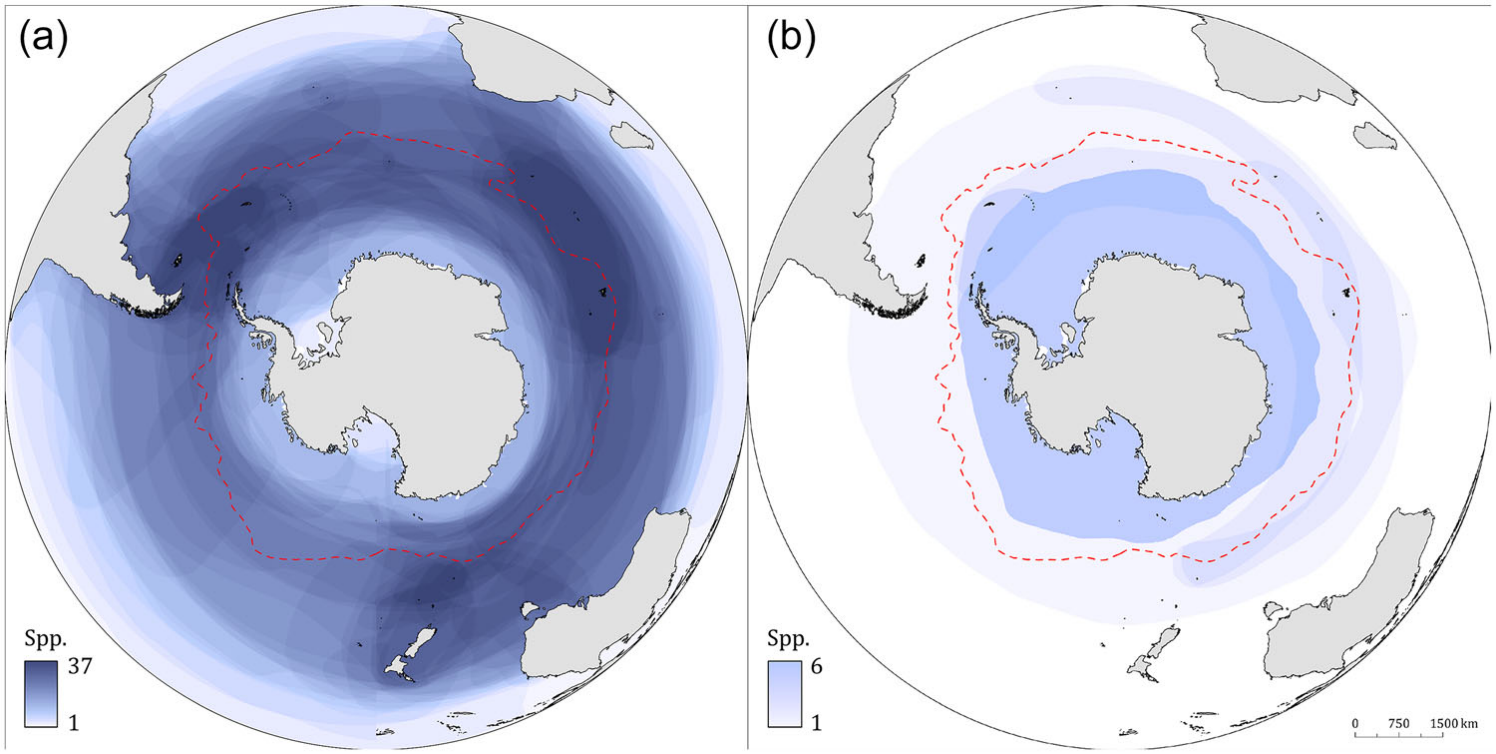
Marine distribution and number of species (BirdLife International, 2024; del Hoyo et al., 1992‐2008;; IUCN, 2023) of (a) seabirds and (b) pinnipeds occurring per location as a measure of the potential for the HPAI H5 virus to spread. The dashed red line indicates the approximate position of the Antarctic Polar Front, based on Moore et al. (1999).

Further expansion to other parts of the continent is plausible given that many Antarctic birds and pinnipeds probably are susceptible to infection and have overlapping ranges that form a wide circumpolar band around Antarctica (BirdLife International, 2024; del Hoyo et al., 1992–2008; IUCN, 2023) (Figure 4). Several seabirds, such as albatrosses, perform circumpolar movements in the Southern Ocean and therefore could support the longitudinal spread of HPAI H5 among Antarctic and sub‐Antarctic islands (e.g., visitors to breeding colonies) or transmission during interactions at sea (e.g., albatrosses and petrels aggregating at foraging sites or near fishing vessels) (Agreement of the Conservation of Albatrosses & Petrels, 2020). This would allow virus expansion along the Antarctic continent and adjacent islands, where dense breeding colonies or other aggregations of susceptible avian or mammalian hosts occur at variable distances of tens to hundreds of kilometers from each other. The success of virus spread depends in part on the distance that infected migrating hosts can travel while actively infected with respect to the distance between colonies (Brown et al., 2008; Ramis et al., 2014; Reperant et al., 2011) and the success of virus transmission to hosts and the amplification of the virus at uninfected colonies, which likely increases with a higher number and density of susceptible hosts at the colony.

Skuas and giant petrels, through their predatory and scavenging behavior, visit numerous sites along the southern tip of South America and in sub‐Antarctic islands and Antarctica. These habits and frequent incursions into breeding colonies of other species suggest these species could also play a significant role in the spread of HPAI H5 among sites in the Antarctic region. Brown skuas, southern fulmars, and kelp gulls have repeatedly been affected on the Falkland Islands and South Georgia and thus far have been among the first species affected in new locations. Predatory and scavenging birds such as great skuas (*Catharacta skua*), gulls, corvids, raptors, and vultures have been involved in HPAI H5 outbreaks in the Northern Hemisphere (Animal and Plant Health Inspection Service, U.S. Department Of Agriculture, 2024; Camphuysen & Gear, 2022; Giacinti et al., 2024; van den Brand et al., 2015; Wunschmann et al., 2024).

## CONSERVATION IMPLICATIONS

The impacts of this HPAI H5 outbreak on wildlife in South America are enormous. First, an immense number of lives have been lost: more than 667,000 wild birds of 83 species and more than 52,000 wild mammals of 11 species reported dead from October 2022 to December 2023. Actual mortality is likely many times larger. This has a direct conservation impact on multiple wild bird and mammal species that are already threatened by other causes. This includes species listed by the International Union for Conservation of Nature as being under threat of extinction in the near future (IUCN, 2023). Based on the number of individuals found dead, seabird species subjected to potential conservation impacts include the Humboldt penguin (vulnerable) and Peruvian pelican, red‐legged cormorant (*Poikilocarbo gaimardi*), sooty shearwater, and elegant tern and Inca tern (all near‐vulnerable). Mammal species subjected to potential conservation impacts include marine otter and southern river otter (*Lontra provocax*) (both endangered) and Chilean dolphin and Burmeister's porpoise (both near threatened) (Appendix S1).

If the HPAI H5 virus spreads further across the Antarctic region, the negative impact on the region's wild bird and mammalian populations could be immense because of their likely susceptibility to mortality from this virus and their occurrence in dense colonies. Based on mortality events that have occurred elsewhere, bird species found in Antarctica and the sub‐Antarctic islands are likely highly susceptible to HPAI H5. Repeated outbreaks in African penguins (*Spheniscus demersus*) in South Africa and Namibia (Molini et al., 2020; Roberts et al., 2023), outbreaks in Humboldt penguins in Peru and Chile (see above), and outbreaks in gentoo penguins on the Falkland Islands and South Georgia (see above) demonstrate that penguins are susceptible to HPAI H5. The recent mortalities of black‐browed albatrosses at the Falkland Islands and of wandering albatrosses in South Georgia confirm that Procellariiformes are also highly susceptible, highlighting the potential conservation risk for other endangered populations of these species. Beyond the avian taxa classically associated with Antarctica, it is likely that the many species of endemic teals, cormorants, and parakeets found on sub‐Antarctic islands are susceptible too (Rijks et al., 2022; Roberts et al., 2023; WAHIS, 2023d, 2023e). The impacts of HPAI H5 in this region will likely be exacerbated by species endemicity and colonial behavior such that vast percentages of the world population are concentrated in a few locations. For example, Steeple Jason Island in the Falkland Islands holds 70% of the world's population of black‐browed albatross. The occurrence of many of these species in dense colonies also likely facilitates rapid virus transmission (Boulinier, 2023). Antarctic and sub‐Antarctic birds already face a myriad of challenges, including declining prey abundance, fisheries bycatch, and climate change. We therefore consider HPAI H5 a major conservation threat to all endemic Antarctic and subantarctic bird species.

Likewise, pinniped populations present in the sub‐Antarctic and Antarctic regions also are probably susceptible to infection and mortality from HPAI H5. All 6 Antarctic pinniped species belong to the families Otariidae or Phocidae (Appendix S2), both of which have been extensively affected by HPAI H5 in South America (Campagna et al., 2023; Leguia et al., 2023). Given the substantial HPAI‐H5‐associated mass mortalities of South American fur seals in southern Brazil and Uruguay, it is probable that the fur seals of the Antarctic region would also be highly susceptible such that catastrophic impacts to the species’ most significant populations may ensue. For example, 95% of the global population of the Antarctic fur seal is concentrated in South Georgia, (IUCN, 2023), and there has been HPAI‐H5‐attributed mortality since October 2023. Similarly, approximately 50% of the global population of the southern elephant seal is concentrated in South Georgia (Boyd et al., 1996), and deaths associated with HPAI H5 have been reported there since October 2023 (Banyard et al., 2024; Government of South Georgia and the South Sandwich Islands, 2023). The most severe HPAI H5 impact in this species so far has been at Peninsula Valdés, Argentina, where 97% of pups died in October and November 2023 (Campagna et al., 2023).

Five of 17 Antarctic cetacean species belong to the families Delphinidae or Phocoenidae (Appendix S2), in which HPAI‐H5‐associated mortality has thus far been detected in Peru and Chile (Chile Servicio Nacional de Pesca y Acuicultura, 2023; Leguia et al., 2023) and in the Northern Hemisphere (Murawski et al., 2024; Thorsson et al., 2023). The mechanisms of transmission of HPAI H5 to cetaceans remain unclear, and no mass mortalities of cetaceans have been attributed to the virus. However, it is plausible that at‐sea mortalities have gone undetected. Therefore, it would be prudent to consider cetaceans as potentially susceptible to HPAI H5 in Antarctica.

Overall, HPAI H5 is a major conservation concern, not just in the short term but also in the long term due to impacts on populations. For example, populations of long‐lived species of birds and mammals could take decades to recover, particularly if HPAI outbreaks recur in consecutive years and the disease kills adult females. Moreover, species currently listed as least concern by the International Union for Conservation of Nature could become threatened, which would have large effects on conservation priorities.

In addition to conservation challenges for directly affected wildlife species, HPAI H5 may have large ecosystem‐level impacts. For example, following Antarctic whaling, the mass removal of animals had substantial ecosystem‐level impacts, including species extinction, breakdown of predatory–prey interactions, modification of nutrient cycling, breakdown in carbon sequestration, which increased CO_2_ emissions and negatively affected global marine productivity, and shifts in species compositions (Herr et al., 2022; Roman et al., 2014). If HPAI H5 were to cause mass mortality events in Antarctica at the scale of those reported in South America, this mass removal of animals from the landscape could have similar, profound impacts on Antarctic coastal and marine ecosystems.

## MANAGING THE ONGOING HPAI H5 OUTBREAK

Now that the HPAI H5 virus has spilled over into wildlife and is no longer dependent on poultry for its continued transmission, there is little that can be done to stop the spread of this virus among free‐living populations. Nevertheless, there are a few actions that may help lower the impact of the ongoing HPAI H5 outbreak on wildlife.

There should be surveillance of wildlife populations for the presence of the HPAI H5 virus based on evidence of unusual morbidity and mortality and virological and serological analyses, including timely sharing of disease diagnosis and viral genome sequences. This will enable prompt detection of new virus introductions and monitoring of virus evolution through phylogenetic analyses, which are relevant for both animal and human health.

There should be a comprehensive recording of mortality events in wildlife and a collection of information and samples to substantiate the cause or causes of mortality. Well‐documented descriptions of HPAI H5 outbreaks in wildlife are required to evaluate the impact of this disease on wildlife populations. Due to its relative remoteness, avian influenza surveillance and investigation of unusual wildlife mortality events require careful advance planning and coordination among scientists from different countries interested in working on avian influenza in Antarctica. This is done through the Antarctic Wildlife Health Network of the Scientific Committee on Antarctic Research (Scientific Committee on Antarctic Research, 2023).

Infected carcasses should be removed as early as possible and repeatedly at wild bird breeding sites that are intensively monitored and managed. Although there are studies that suggest that carcass removal effectively reduces contamination and transmission to other animals (Knief et al., 2024; Reperant et al., 2021; Rijks et al., 2022; Yamamoto et al., 2017), efficacy in different scenarios remains uncertain. In Antarctica, there are additional challenges associated with carcass removal due to rules preventing carcass burial and the lack of incinerators (Dewar et al., 2023). Also, the potential benefits of removing carcasses need to be weighed against the potential adverse effects of repeated disturbance of breeding colonies.

Nonessential human activities (e.g., tourism, extraction/exploitation of natural resources) should be reduced at affected sites to prevent the unintentional spread of the virus and to minimize the risk of human exposure. Rules for tourism are of particular relevance to Antarctica, where there were 122,072 tourists in the 2023–2024 austral season (International Association of Antarctica Tour Operators, 2024). These measures may also be particularly important at breeding sites of affected birds and mammal species to reduce disturbance and enhance postoutbreak population recovery.

Essential human activities (e.g., research, implementation of conservation measures) at affected sites should involve appropriate biosafety measures, such as disinfection of footwear and the use of personal protective equipment. Some measures need to be adapted to the unique circumstances in the sub‐Antarctic and Antarctica (Dewar et al., 2023). This aims both to reduce the risk of human‐caused spread of the virus to other wildlife populations and to protect people exposed to the HPAI H5 virus from infected animals.

Under no circumstance should disease control measures include killing wild birds or mammals, spraying toxic products, or actions that negatively affect wetlands and other habitats, or deterring animals from access to their habitat. This is based on the advice of the Food and Agriculture Organization and the WOAH and international obligations under the Convention on Migratory Species, the Agreement on the Conservation of African–Eurasian Migratory Waterbirds, and the Ramsar Convention on Wetlands (CMS & FAO, 2023).

## CONCLUDING REMARKS

A consequence of the Anthropocene is an increase in the rate of emerging infectious disease events. These events include diseases spilling over from farmed or traded animals into free‐ranging wildlife populations, such as mycoplasmosis from poultry to North American songbirds (Dhondt et al., 2005), African swine fever from domestic pigs to Eurasian wild boar and Asian wild pigs (Luskin et al., 2020), and HPAI H5 from poultry to multiple species of wild birds and mammals worldwide (Wille & Barr, 2022). This is a paradigm shift where wildlife is not just a source but also a victim of emerging infectious diseases (Kuiken & Cromie, 2022).

A parallel paradigm shift is needed in infectious disease prevention and control to prevent the escalation of emerging infectious disease events in wildlife, livestock, and humans from happening and to minimize their impacts when they do occur. To this end, we advocate a one‐health approach, which aims to optimize the health not only of people and livestock but also of wildlife and ecosystems. Such a one‐health‐based paradigm shift includes integrating previously siloed government departments of agriculture, public health, and the environment; building a one‐health workforce capable of handling these complex wicked problems; and greatly increasing financial support for research, surveillance, and management of emerging infectious disease events in wildlife and ecosystems to levels similar to those for livestock and people.

The current HPAI H5 outbreak, which stems from a virus that emerged in a rapidly expanding poultry sector in eastern Asia, has led to catastrophic impacts on seabird and pinniped populations in South America. This highlights that ecosystems are globally connected, that viruses do not recognize political or species barriers, and that once such an adaptable virus spills over into wildlife, it is out of human control. To prevent future HPAI outbreaks in wildlife, reduce risk for humans, and protect food security, the links between livestock production, wildlife populations, and ecosystem functioning need to be considered in disease prevention, preparedness, and response. Moreover, the drivers of disease emergence must be addressed proactively and with a renewed focus on biodiversity conservation.

## Supporting information



Supporting Information

Supporting Information
