# Supplementary material for: Emergence, spread, and impact of high‐pathogenicity avian influenza H5 in wild birds and mammals of South America and Antarctica
Source: Conserv Biol. 2025 May 31;40(1):e70052. doi: 10.1111/cobi.70052 (PMC12856802; doi:10.1111/cobi.70052)
Supplement: Supplementary file 1 — Supporting Information [file COBI-40-e70052-s001.docx]

**Appendix S1: Number of birds and mammals reported dead from 1 November 2022 to 10th of December 2023 by countries in South America.**

Asterisks indicate species-country associations where HPAI H5 virus infection was not confirmed by at least one case. Numerical comparisons among countries may be unreliable since countries differ considerably in their approach for surveillance, diagnostic methods and reporting of suspected/confirmed HPAI cases.

|  |  | |  |  | **Number of individuals reported dead per country^4^** | | | | | | | | | |
| --- | --- | --- | --- | --- | --- | --- | --- | --- | --- | --- | --- | --- | --- | --- |
| **Family / Common name /Species name** | | | **IUCN Red List assessment** | **Estimated global population size** | **Bolivia** | **Peru** | **Chile** | **Argentina** | **Ecuador** | **Brazil** | **Uruguay** | **Colombia** | **Venezuela** | **FMI^5^** |
| **Birds** | |  |  |  |  |  |  |  |  |  |  |  |  |  |
| Accipitridae | |  |  |  |  |  |  |  |  |  |  |  |  |  |
|  | Great black hawk | *Buteogallus urubitinga* | Least concern | 500,000-4,999,999^3^ |  |  |  |  |  | 1 |  |  |  |  |
|  | Black-chested buzzard-eagle | *Geranoaetus melanoleucus* | Least concern | unknown^1,3^ |  | 13 | 1 |  |  |  |  |  |  |  |
|  | Harris's hawk | *Parabuteo unicinctus* | Least concern | unknown^1,3^ |  |  | 2 |  |  |  |  |  |  |  |
| Anatidae | |  |  |  |  |  |  |  |  |  |  |  |  |  |
|  | White-cheeked pintail | *Anas bahamensis* | Least concern | 177,000 – 1,080,000^2^ |  | 1 |  | 3 |  |  |  |  |  |  |
|  | Yellow-billed teal | *Anas flavirostris* | Least concern | 1,043,000 – 1,133,000^2^ |  |  | 1 |  |  |  |  |  |  |  |
|  | Yellow-billed pintail | *Anas georgica* | Least concern | 100,000 – 1,000,000^2^ |  |  | 1 |  |  |  |  |  |  |  |
|  | Black-necked swan | *Cygnus melancoryphus* | Least concern | 25,000 – 100,000^2^ |  |  | 107 | 21 |  | 1 | 142 |  |  |  |
|  | Black-bellied whistling-duck | *Dendrocygna autumnalis* | Least concern | 200,000-2,000,000^2^ |  |  |  |  |  |  |  | 1 |  |  |
|  | White-faced whistling-duck | *Dendrocygna viduata* | Least concern | 2,100,000 – 2,500,000^2^ |  |  |  |  |  |  |  | 1 |  |  |
|  | Chiloé wigeon | *Mareca sibilatrix* | Least concern | 100,000 – 1,000,000^2^ |  |  | 1 |  |  |  |  |  |  |  |
|  | Silver teal | *Spatula versicolor* | Least concern | 50,000 – 200,000^2^ |  |  |  | 1 |  |  |  |  |  |  |
|  | Andean goose | *Chloephaga melanoptera* | Least concern | 25,000 – 100,000^2^ |  | 102 |  | 2 |  |  |  |  |  |  |
|  | Coscoroba swan | *Coscoroba coscoroba* | Least concern | 10,000 - 25,000^2^ |  |  | 10 |  |  |  |  |  |  |  |
|  | Magellanic steamer duck | *Tachyeres pteneres* | Least concern | 10,000 - 100,000^2^ |  |  | 3 |  |  |  |  |  |  |  |
|  | Upland goose | *Chloephaga picta* | Least concern | 188,000 – 405,000^2^ |  |  | 5 |  |  |  |  |  |  |  |
| Ardeidae | |  |  |  |  |  |  |  |  |  |  |  |  |  |
|  | Great white egret | *Ardea alba* | Least concern | 1,625,000-2,410,000^2^ |  |  | 1 |  |  |  |  |  |  |  |
|  | Cattle egret | *Bubulcus ibis* | Least concern | 3,030,801 – 9,757,000^2^ |  | 1 |  |  |  |  |  |  |  |  |
|  | Snowy egret | *Egretta thula* | Least concern | 713,000 – 2,428,000^2^ |  | 1 |  |  |  | 1 |  |  |  |  |
|  | Black-crowned night-heron | *Nycticorax nycticorax* | Least concern | 588,300 – 2,935,000^2^ |  |  | 1 |  |  |  |  |  |  |  |
| Cathartidae | |  |  |  |  |  |  |  |  |  |  |  |  |  |
|  | Turkey vulture | *Cathartes aura* | Least concern | unknown^1,3^ |  |  | 5 |  |  |  |  |  |  |  |
|  | American black vulture | *Coragyps atratus* | Least concern | unknown^1,3^ |  | 1 | 2 |  |  |  |  |  |  |  |
|  | Andean condor | *Vultur gryphus* | Vulnerable | 6,700^3^ |  |  |  |  |  |  |  |  |  |  |
| Charadriidae | |  |  |  |  |  |  |  |  |  |  |  |  |  |
|  | American golden plover | *Pluvialis dominica* | Least concern | 395,000 – 605,000^2^ |  |  |  |  |  | 1 |  |  |  |  |
|  | Southern lapwing | *Vanellus chilensis* | Least concern | 2,000,000 – 2,100,000^2^ |  |  | 1 |  |  |  |  |  |  |  |
| Diomedeidae | |  |  |  |  |  |  |  |  |  |  |  |  |  |
|  | Waved albatross | *Phoebastria irrorata* | Critically endangered | >35,000^3^ |  | 3 |  |  |  |  |  |  |  |  |
|  | Black-browed albatross | *Thalassarche melanophris* | Least concern | 1,400,000^1^ |  |  | 1 |  |  |  |  |  |  | 1 |
| Falconidae | |  |  |  |  |  |  |  |  |  |  |  |  |  |
|  | Crested caracara | *Caracara plancus* | Least concern | 2,500,000-  4,999,999^3^ |  | 28 |  |  |  |  |  |  |  |  |
|  | Peregrine falcon | *Falco peregrinus* | Least concern | 100,000-499,999^3^ |  | 2 | 3 |  |  |  |  |  |  |  |
|  | Chimango caracara | *Phalcoboenus chimango* | Least concern | Unknown^1,3^ |  |  | 3 |  |  |  |  |  |  |  |
| Fregatidae | |  |  |  |  |  |  |  |  |  |  |  |  |  |
|  | Magnificent frigatebird | *Fregata magnificens* | Least concern | 130,000^3^ |  | 12 |  |  | 6000 | 1 |  |  |  |  |
|  | Great frigatebird | *Fregata minor* | Least concern | 16,700^2^ |  |  |  |  | 1002 |  |  |  |  |  |
| Haematopodidae | |  |  |  |  |  |  |  |  |  |  |  |  |  |
|  | Blackish oystercatcher | *Haematopus ater* | Least concern | 32,000 – 134,000^2^ |  |  | 50 |  |  |  |  |  |  |  |
|  | American oystercatcher | *Haematopus palliatus* | Least concern | 36,500 – 112,000^2^ |  | 2 | 1 |  |  |  |  |  |  |  |
| [Hirundidinae](https://www.iucnredlist.org/search?taxonomies=22673336&searchType=species) | |  |  |  |  |  |  |  |  |  |  |  |  |  |
|  | Blue-and-white swallow | *Pygochelidon cyanoleuca* | Least concern | 5,000,000-  50,000,000^3^ | 4 |  |  |  |  |  |  |  |  |  |
| Laridae | |  |  |  |  |  |  |  |  |  |  |  |  |  |
|  | Inca tern | *Larosterna inca* | Near threatened | 150,000^2^ |  | 7987 | 239 |  |  |  |  |  |  |  |
|  | Belcher's gull | *Larus belcheri* | Least concern | 1 – 10,000^2^ |  | 1063 | 293 |  |  |  |  |  |  |  |
|  | Grey-headed gull | *Larus cirrocephalus* | Least concern | 250,000 – 540,000^2^ |  |  |  |  | 1 | 2 |  |  |  |  |
|  | Kelp gull | *Larus dominicanus* | Least concern | 3,287,000 – 4,290,000^2^ |  |  | 4594 | 1 |  |  |  |  |  |  |
|  | Brown-hooded gull | *Larus maculipennis* | Least concern | 100,000 – 1,000,000^2^ |  |  | 48 |  |  | 1 |  |  |  |  |
|  | Grey gull | *Larus modestus* | Least concern | 25,000^2^ |  |  | 1016 |  |  |  |  |  |  |  |
|  | Franklin's gull | *Larus pipixcan* | Least concern | 1,000,000 – 1,490,000^2^ |  |  | 95 |  |  |  |  |  |  |  |
|  | Dolphin gull | *Larus scoresbii* | Least concern | 9,000-28,000^2^ |  |  | 2 |  |  |  |  |  |  |  |
|  | Black skimmer | *Rynchops niger* | Least concern | 125,000 – 208,000^2^ |  |  | 26 |  |  |  |  |  |  |  |
|  | Royal tern | *Thalasseus maximus* | Least concern | 370,000 – 380,000^2^ |  |  |  | 2400 |  | 60 | 1 |  |  |  |
|  | Cabot's/Cayenne tern | *Thalasseus acuflavidus* | Least concern | 153,000 - 158,000^2^ |  |  |  |  |  | 858 |  |  |  |  |
|  | South American tern | *Sterna hirundinacea* | Least concern | 25,000 – 1,000,000^2^ |  |  | 58 |  |  |  | 5 |  |  |  |
|  | Elegant tern | *Thalasseus elegans* | Near threatened | 270,000^2^ |  |  | 135 |  |  |  |  |  |  |  |
|  | Common tern | *Sterna hirundo* | Least concern | 2,260,000 – 3,950,300^2^ |  |  |  |  |  | 15 |  |  |  |  |
| Passeridae | |  |  |  |  |  |  |  |  |  |  |  |  |  |
|  | House sparrow | *Passer domesticus* | Least concern | 896,000,000-  1,310,000,000^3^ |  |  | 1 |  |  |  |  |  |  |  |
| Pelecanidae | |  |  |  |  |  |  |  |  |  |  |  |  |  |
|  | Brown pelican | *Pelecanus occidentalis* | Least concern | 345,000 – 400,000^2^ |  |  |  |  |  |  |  | 302 | 173 |  |
|  | Peruvian pelican | *Pelecanus thagus* | Near threatened | 100,000 - 1,000,000^2^ |  | 57447 | 4509 |  |  |  |  |  |  |  |
| Phalacrocoracidae | |  |  |  |  |  |  |  |  |  |  |  |  |  |
|  | Imperial shag | *Leucocarbo atriceps* | Least concern | 333,000 - 1.360.000^2^ |  |  | 7 |  |  |  |  |  |  |  |
|  | Guanay cormorant | *Leucocarbo bougainvilliorum* | Near threatened | 2,500,00 – 5,000,000^2^ |  | 254793 | 6380 |  |  |  |  |  |  |  |
|  | Neotropical cormorant | *Nannopterum brasilianus* | Least concern | 2,160,000 – 3,150,000^2^ |  |  | 726 |  |  | 1 |  |  |  |  |
|  | Red-legged cormorant | *Poikilocarbo gaimardi* | Near threatened | 30,000^2^ |  |  | 498 |  |  |  |  |  |  |  |
|  | Rock shag | *Leucocarbo magellanicus* | Least concern | 106,000 – 202,000^2^ |  |  | 1 |  |  |  |  |  |  |  |
| Phoenicopteridae | |  |  |  |  |  |  |  |  |  |  |  |  |  |
|  | Chilean flamingo | *Phoenicopterus chilensis* | Near threatened | 290,000^2^ |  | 3 |  |  |  |  |  |  |  |  |
|  | Puna flamingo | *Phoenicoparrus jamesi* | Near threatened | 106,000 – 107,000^2^ |  |  |  | 237 |  |  |  |  |  |  |
| Podicipedidae | |  |  |  |  |  |  |  |  |  |  |  |  |  |
|  | Great grebe | *Podiceps major* | Least concern | 40,000 – 140,000^2^ |  |  | 3 |  |  |  |  |  |  |  |
| Procellariidae | |  |  |  |  |  |  |  |  |  |  |  |  |  |
|  | Sooty shearwater | *Ardenna grisea* | Near threatened | 8,800,000^1^ |  | 6 | 304 |  |  |  |  |  |  |  |
|  | Southern fulmar | *Fulmarus glacialoides* | Least concern | 4,000,000^1^ |  |  |  |  |  |  |  |  |  | 2 |
|  | Southern giant petrel | *Macronectes giganteus* | Least concern | 95,600-108,000^1^ |  |  | 7 |  |  |  |  |  |  |  |
|  | Antarctic prion | *Pachyptila desolata* | Least concern | 50,000,000^1^ |  |  |  |  |  | 1 |  |  |  |  |
|  | Peruvian diving petrel | *Pelecanoides garnotii* | Near threatened | 100,000^1^ |  |  | 25 |  |  |  |  |  |  |  |
|  | White-chinned petrel | *Procellaria aequinoctialis* | Vulnerable | 3,000,000^1^ |  |  |  |  |  | 1 |  |  |  |  |
|  | Manx shearwater | *Puffinus puffinus* | Least concern | 680,000-790,000^1^ |  |  |  |  |  | 3 |  |  |  |  |
| Psittacidae | |  |  |  |  |  |  |  |  |  |  |  |  |  |
|  | Slender-billed parakeet | *Enicognathus leptorhynchus* | Least concern | Unknown^1,3^ |  |  | 14 |  |  |  |  |  |  |  |
| Rallidae | |  |  |  |  |  |  |  |  |  |  |  |  |  |
|  | Red-gartered coot | *Fulica armillata* | Least concern | 1,000,000^2^ |  |  | 2 |  |  |  |  |  |  |  |
| Recurvirostridae | |  |  |  |  |  |  |  |  |  |  |  |  |  |
|  | White-backed stilt | *Himantopus melanurus* | not classified | 100,000 - 1,000,000^2^ |  |  | 1 |  |  |  |  |  |  |  |
| Scolopacidae | |  |  |  |  |  |  |  |  |  |  |  |  |  |
|  | Ruddy turnstone | *Arenaria interpres* | Least concern | 460,000-700,000^2^ |  | 1 |  |  |  |  |  |  |  |  |
|  | Sanderling | *Calidris alba* | Least concern | 705,000 – 780,000^2^ |  | 1 | 12 |  |  |  |  |  |  |  |
|  | Whimbrel | *Numenius phaeopus* | Least concern | 1,800,000 – 2,650,000^2^ |  | 1 | 2 |  |  |  |  |  |  |  |
|  | Lesser yellowlegs | *Tringa flavipes* | Least concern | 400,000^2^ |  |  | 1 |  |  |  |  |  |  |  |
| Spheniscidae | |  |  |  |  |  |  |  |  |  |  |  |  |  |
|  | Humboldt penguin | *Spheniscus humboldti* | Vulnerable | 23,800^1^ |  | 371* | 3721 |  |  |  |  |  |  |  |
| Stercorariidae | |  |  |  |  |  |  |  |  |  |  |  |  |  |
|  | Brown skua | *Catharacta antarctica* | Least concern | 26,000-28,000^1^ |  | 25 |  |  |  |  |  |  |  |  |
|  | Chilean skua | *Catharacta chilensis* | Least concern | 2,500-9,999^1^ |  |  | 6 |  |  |  |  |  |  |  |
| Strigidae | |  |  |  |  |  |  |  |  |  |  |  |  |  |
|  | Tropical screech-owl | *Megascops choliba* | Least concern | 500,000-  4,999,999^1^ |  |  |  |  |  | 1 |  |  |  |  |
| Sulidae | |  |  |  |  |  |  |  |  |  |  |  |  |  |
|  | Brown booby | *Sula leucogaster* | Least concern | 200,000^1^ |  |  |  |  |  | 3 |  |  |  |  |
|  | Blue-footed booby | *Sula nebouxii* | Least concern | 90,000^1^ |  | 4 |  |  | 3 |  |  |  |  |  |
|  | Red-footed booby | *Sula sula* | Least concern | 1,400,000^1^ |  |  |  |  | 6 |  |  |  |  |  |
|  | Peruvian booby | *Sula variegata* | Least concern | 1,200,000^1^ |  | 235643 | 6506 |  |  |  |  |  |  |  |
| **Mammals** | |  |  |  |  |  |  |  |  |  |  |  |  |  |
| Delphinidae | |  |  |  |  |  |  |  |  |  |  |  |  |  |
|  | Chilean dolphin | *Cephalorhynchus eutropia* | Near threatened | unknown |  |  | 1 |  |  |  |  |  |  |  |
|  | Short-beaked common dolphin | *Delphinus delphis* | Least concern | unknown |  | 1 | 9* |  |  |  |  |  |  |  |
|  | Dusky dolphin | *Lagenorhynchus obscurus* | Least concern | unknown |  |  | 28 |  |  |  |  |  |  |  |
| Phocoenidae | |  |  |  |  |  |  |  |  |  |  |  |  |  |
|  | Burmeister's porpoise | *Phocoena spinipinnis* | Near threatened | unknown |  |  | 36 |  |  |  |  |  |  |  |
| Mustelidae | |  |  |  |  |  |  |  |  |  |  |  |  |  |
|  | Marine otter | *Lontra felina* | Endangered | unknown |  |  | 43 |  |  |  |  |  |  |  |
|  | Southern river otter | *Lontra provocax* | Endangered | unknown |  |  | 1 |  |  |  |  |  |  |  |
| Otariidae | |  |  |  |  |  |  |  |  |  |  |  |  |  |
|  | South American fur seal | *Arctocephalus australis* | Least concern | 109,500**^3^** |  |  | 27* | 13 |  | 552 | 800 |  |  |  |
|  | South American sea lion | *Otaria byronia* | Least concern | 222,500**^3^** |  | 10457 | 20070 | 1367 |  |  |  |  |  |  |
| Phocidae | |  |  |  |  |  |  |  |  |  |  |  |  |  |
|  | Southern elephant seal | *Mirounga leonina* | Least concern | 325,000**^3^** |  |  | 16* | 17400 |  |  |  |  |  |  |
| Procyonidae | |  |  |  |  |  |  |  |  |  |  |  |  |  |
|  | South American coati | *Nasua nasua* | Least concern | unknown |  |  |  |  |  |  | 16 |  |  |  |

^1^Population estimate (number of mature individuals) based on data from BirdLife International’s “IUCN Red List for birds”. Downloaded from <https://datazone.birdlife.org> in March 2024.

^2^Population estimate (total population) based on data from Wetlands International’s “Waterbird Populations Portal”. Retrieved from wpp.wetlands.org in March 2024.

^3^Population estimate (number of mature individuals) based on data from IUCN’s “The IUCN Red List of Threatened Species”. Version 2023-1. Retrieved from https://www.iucnredlist.org>lkjd in March 2024.

^4^Numbers of individuals reported dead per country based on data from the following sources:

- Anonymous (2023). Preocupación por 552 lobos marinos muertos a pocos kilómetros del Chuy [Concern over 552 dead sea lions a few kilometers from Chuy] [last accessed 10 December 2023]. Available from: [www.elobservador.com.uy/nota/preocupacion-por-552-lobos-marinos-muertos-a-pocos-kilometros-del-chuy-20231025183255](http://www.elobservador.com.uy/nota/preocupacion-por-552-lobos-marinos-muertos-a-pocos-kilometros-del-chuy-20231025183255)
- Argentina Servicio Nacional de Sanidad y Calidad Agroalimentaria (2023). Estado de la situación epidemiológica en la Argentina [State of the epidemiological situation in Argentina] [cited 2023 10 December]. Available from: [www.argentina.gob.ar/senasa/estado-de-la-situacion-epidemiologica-en-la-argentina](http://www.argentina.gob.ar/senasa/estado-de-la-situacion-epidemiologica-en-la-argentina)
- Banyard, A. C., Bennison, A., Byrne, A. M. P., Reid, S. M., Lynton-Jenkins, J. G., Mollett, B., De Sliva, D., Peers-Dent, J., Finlayson, K., Hall, R., Blockley, F., Blyth, M., Falchieri, M., Fowler, Z., Fitzcharles, E. M., Brown, I. H., & James, J. (2024). Detection and spread of high pathogenicity avian influenza virus H5N1 in the Antarctic Region. Nat. Commun., 15(1). doi.org/doi.org/10.1038/s41467-024-51490-8
- Brazil Ministério da Agricultura e Pecuária (2023). Influenza Aviária [Avian influenza] [last accessed 10 December 2023]. Available from: <https://mapa-indicadores.agricultura.gov.br/publico/extensions/SRN/SRN.html>
- Campagna C, Uhart M, Falabella V, Campagna J, Zavattieri V, Vanstreels RET, Lewis M (2024). Catastrophic mortality of southern elephant seals caused by H5N1 avian influenza. Marine Mammal Science, 40: 322.
- Chile Servicio Agrícola y Ganadero (2023). Influenza Aviar (IA) [Avian influenza dashboard] [last accessed 10 December 2023]. Available from: [www.sag.gob.cl/ambitos-de-accion/influenza-aviar-ia](http://www.sag.gob.cl/ambitos-de-accion/influenza-aviar-ia)
- Chile Servicio Nacional de Pesca y Acuicultura (2023). Influenza Aviar [Avian influenza summary] [last accessed 10 December2023]. Available from: [www.sernapesca.cl/influenza-aviar](http://www.sernapesca.cl/influenza-aviar)
- Falkland Islands Department of Agriculture (2023). Avian influenza information [last accessed December 2023]. Available from: [www.falklands.gov.fk/agriculture/avian-influenza](http://www.falklands.gov.fk/agriculture/avian-influenza)
- Peru Ministerio de Salud (2023). Sala de influenza aviar [Avian influenza dashboard] [Last accessed 10 December 2023]. Available from: [www.dge.gob.pe/influenza-aviar-ah5/#aves](http://www.dge.gob.pe/influenza-aviar-ah5/#aves)
- Uhart, M., Vanstreels, R. E. T., Nelson, M. I., Olivera, V., Campagna, J., Zavattieri, V., Lemey, P., Campagna, C., Falabella, V., & Rimondi, A. (2024). Massive outbreak of Influenza A H5N1 in elephant seals at Península Valdés, Argentina: increased evidence for mammal-to-mammal transmission. bioRxiv, 2024.2005.2031.596774. https://doi.org/10.1101/2024.05.31.596774
- Vara D, Mano A (2023). Bird flu kills over 900 seals, sea lions in south Brazil [last accessed 14 December 2023]. Available from: [www.reuters.com/world/americas/bird-flu-kills-over-900-seals-sea-lions-south-brazil-2023-12-11/](http://www.reuters.com/world/americas/bird-flu-kills-over-900-seals-sea-lions-south-brazil-2023-12-11/)
- World Organisation for Animal Health. World Animal Health Information System (2023) [last accessed 15 December 2023]. Available from: wahis.woah.org/

^5^FMI: Falklands (Malvinas) Islands
